# Clinical application of health education based on the “Internet+perioperative health education” mode for patients undergoing surgery for anterior cruciate ligament injury

**DOI:** 10.12669/pjms.41.8.10997

**Published:** 2025-08

**Authors:** Ao Xiong, Tao Liao, Zongming Bao, Xingyue Chen

**Affiliations:** 1 Ao Xiong Department of Operating Theatre, Sichuan Province Orthopedic Hospital, Chengdu 610041, Sichuan, China; 2 Tao Liao Department of Operating Theatre, Sichuan Province Orthopedic Hospital, Chengdu 610041, Sichuan, China; 3 Zongming Bao Department of Operating Theatre, Sichuan Province Orthopedic Hospital, Chengdu 610041, Sichuan, China; 4 Xingyue Chen Department of Operating Theatre, Sichuan Province Orthopedic Hospital, Chengdu 610041, Sichuan, China

**Keywords:** Internet+, Perioperative health education, Surgery for anterior cruciate ligament injury, Application effect

## Abstract

**Objective:**

To explore the effect of “Internet+ perioperative health education” on perioperative health education for patients undergoing surgery for anterior cruciate ligament (ACL) injury.

**Methods:**

This retrospective study included 120 patients with ACL injury treated in Sichuan Province Orthopedic Hospital from April 2021 to April 2023.They were divided into the control group and the observation group according to the way of education. The control group received routine perioperative health education. The observation group adopted the model of “Internet plus perioperative health education”. Outcomes included Self Rating Anxiety Scale (SAS), physiological indicators (heart rate, blood pressure), psychological care effects, and satisfaction.

**Results:**

Post-education, the SAS scores of both groups of patients decreased compared to before education(p<0.05), and the reduction in SAS scores was greater in the observation group(p<0.05); The heart rate and blood pressure indicators of the observation group patients were more stable(p<0.05).The perioperative psychological care effect of the observation group patients was better than that of the control group, and the postoperative satisfaction was higher than that of the control group (p<0.05).

**Conclusion:**

Carrying out “Internet plus perioperative health education” for patients undergoing anterior cruciate ligament injury surgery can effectively improve their perioperative anxiety, stabilize their psychological state, improve their perioperative physiological indicators, and improve their postoperative satisfaction

## INTRODUCTION

The anterior cruciate ligament (ACL) originates from the posterior part of the medial surface of the lateral femoral condyle, and attaches to the anterior part of the intercondylar spine of the tibial plateau through the joint cavity in the forward, distal, and inward directions.^1^ ACL is a strip-shaped connective tissue that connects the intercondylar spine of the tibia and the medial surface of the lateral condyle of the femur. As an important structure stabilizing the knee joint, the ACL can resist forward tibial moving loads and rotational loads.

There may be a risk of ACL injury when the knee joint is subjected to external forces from the anterior or lateral side during flexion. With the popularization of modern fitness activities, an increasing number of people are participating in sports, resulting in a gradual increase in the number of patients suffering from knee joint injuries annually. In the absence of proper treatment, prolonged injury can cause knee joint degeneration, worsen knee joint dysfunction, and seriously disturb patients’ work and quality of life.^2^ It has been reported that early ACL reconstruction is crucial for restoring knee joint stability, and preventing, delaying, and reducing secondary intra-articular injuries.^3^,^4^

Furthermore, the rapid development of arthroscopic technique boosts the application of arthroscopic ACL reconstruction, as recommended by increasingly more scholars.^5^ Despite relatively minor trauma, arthroscopic ACL reconstruction is still accompanied by several disadvantages, such as a longer postoperative recovery period, and higher incidence of postoperative complications. Significantly, comprehensive perioperative health education for patients with ACL injury can enhance their awareness of relevant knowledge, promote their active cooperation with surgery, alleviate their anxiety caused by insufficient perioperative knowledge, decrease perioperative complications, and improve patient satisfaction. It has become an important part of perioperative care for patients with ACL injury during diagnosis and treatment.

Health education intends to study and publicize health knowledge and technology, adjust individual and group behaviors, prevent diseases, eliminate risk factors, and promote human health. In particular, perioperative health education is of great significance for the perioperative recovery of patients.^6^ Traditional perioperative health education, in the form of on-site communication mainly, is generally carried out by nursing staff through oral education, distribution of promotional brochures, or watching video lectures, broadcasts, etc.

However, the implementation of perioperative health education is still formalistic owing to the delayed development of holistic nursing in the operating room and insufficient staffing of operating room nurses.^7^ Critically, the “Internet+” mode can transform traditional textual or oral teaching content into specific content. It enables the introduction of perioperative health education knowledge to patients through text, images, animations, videos, etc., which can enhance patients’ continuous learning and improve their self-awareness of the disease.^8^,^9^

In this regard, this study applied the “Internet+ perioperative health education” mode in perioperative health education of patients undergoing ACL reconstruction, aiming to increase patients’ awareness of ACL injury and their understanding of perioperative precautions. By observing whether the proposed method can better improve the effect of perioperative health education for this type of surgery, findings in this study may offer additional strategies for perioperative education of patients undergoing ACL reconstruction.

## METHODS

This was a retrospective study. One hundred and twenty patients who were hospitalized in the Orthopedic Department of Sichuan Province Orthopedic Hospital for arthroscopic ACL reconstruction from April 2021 to April 2023. The included patients were divided into the control group and the observation group according to the way of education, with 60 patients in each group. All surgeries were performed by the same surgical team consisting of three senior orthopedic surgeons (each with >100 ACL reconstructions annually) using standardized arthroscopic techniques.

### Ethical Approval:

This study was approved by the Institutional Ethics Committee of Sichuan Province Orthopedic Hospital (No.: KY2020-026-01; Date: July 20, 2020), and written informed consent was obtained from all participants.

### Inclusion criteria:

- Patients with ACL complete rupture by MRI, positive for drawer test and Lachman test after admission.
- Who met the diagnostic criteria for ACL rupture and underwent arthroscopic ACL reconstruction.
- Without hearing, language expression, or cognitive impairments.
- Patients themselves or their families had social media accounts and devices.
- Who voluntarily participated in the study and were informed of relevant details.
- Those who could communicate well with researchers and complete the study according to relevant requirements.

### Exclusion criteria:

- Patients with other serious diseases.
- Who had the surgery canceled.
- Non-Internet users.
- With cognitive impairment.
- Those who were unwilling or unable to cooperate with the study.

### Health education:

Patients in the control group were given routine preoperative health education by operating room nurses with >5 years of working experience one day prior to the surgery. Specifically, perioperative precautions and surgical instructions were explained to patients via one-to-one oral communication, combined with the distribution of preoperative health education and guidance leaflets, and answering questions from patients. Regular postoperative follow-up was conducted on the first day after surgery.

Patients in the observation group were provided with whole-process “Internet+ perioperative health education” by operating room nurses with >5 years of working experience. One day before the operation, health education was conducted during preoperative visits to guide patients to watch special videos on the Internet (e.g., operating instructions, operating room environment and the process of entering the operating room), timely answer patients’ and family members’ questions, and offer Internet-based continuous nursing.

### Observation indexes:

The anxiety of patients in both groups was evaluated using the Self-Rating Anxiety Scale (SAS)^10^ before perioperative health education and anesthesia induction. Patients’ physiological indicators such as heart rate, and diastolic and systolic blood pressure were monitored before and after health education, as well as before anesthesia induction. The effect of perioperative psychological care using the Perioperative Psychological Care Effect Evaluation Table developed by Jiao Y^11^ from the time patients entered the operating room to 72 hours after surgery. In addition, patients were followed up 2-3 days after surgery to confirm patient satisfaction. Double data entry by two researchers. All patients were followed up for six months after surgery, and their long-term satisfaction and complications were evaluated through monthly WeChat surveys and outpatient visits. Using a 4-point Likert scoring method, the frequency of symptom occurrence was graded in SAS, with a total of 20 questions. Patient status of anxiety was negatively correlated with the total score. The total score ranged from 0 to 80 points, and the standard score was calculated by multiplying the total score by 1.25. With 50 points as the threshold, patients scored 50-59, 60-69 and ≥70 points were classified as mild, moderate and severe anxiety, respectively.^10^

The effect of perioperative psychological care was evaluated using the Perioperative Psychological Care Effect Evaluation Table.^11^ There were nine items, including blood pressure, pulse, facial expression, self-perception, muscle tension, intraoperative pain response, postoperative pain response, postoperative emotions, and out-of-bed activity. Each item was divided into 3 levels, and rated 0 point, one point and two points, with a maximum score of 18 points. There were three levels based on the scores, i.e., dissatisfied (0-6 points), relatively satisfied (7-11 points), and satisfied (12-18 points). Each item was scored from the time patients entered the operating room until 72 hours after surgery to generate the cumulative score for inter-group comparison of the total scores.

Satisfaction questionnaires were distributed to collect postoperative information from patients. The questionnaire consisted of 10 items, and patients’ satisfaction degrees with perioperative nursing included very satisfied, satisfied, and dissatisfied (specific reasons required). The satisfaction rate was calculated based on the formula of “the satisfaction rate = (very satisfied cases+satisfied cases)/total cases×100%.

### Statistical analysis:

By using SPSS23.0 software for statistical analysis, measurement data in the form of (`x±s) and counting data in proportion (%) were respectively, inter-group comparisons used independent samples t-test for measurement data and χ² test for categorical variables. The presence of statistical difference was confirmed when P<0.05.

## RESULTS

No statistical difference was observed in the comparison of general data between groups (P>0.05), indicating the existence of comparability between the two groups(Table-I).

**Table-I:** Comparison of general data between groups.

Groups	Cases (n)	Gender (n)		Age (*χ̅*±*S*, years)	BMI (*χ̅*±*S*, kg/m^2^)
		Male	Female		
Control group	60	37	23	41.04±6.95	23.71±4.39
Observation group	60	35	25	37.62±7.72	24.16±4.57
*χ*^2^/*t*		0.081		1.024	0.235
*P*		0.792		0.326	0.717

Before health education, both groups of patients were in a moderate state of anxiety (SAS score of 60-69), showing no statistically significant difference between groups (P>0.05). Before anesthesia induction, both groups of patients showed a decrease in SAS score compared to that before health education; meanwhile, inter-group comparison revealed that the observation group had a more significant reduction in SAS score than the control group, with statistically significant differences (P<0.05)(Table-II).

**Table II:** Comparison of SAS scores between groups before the implementation of health education and before anesthesia induction (*χ̅*±*S*).

Groups	Before health education	Before anesthesia induction
Control group (n=60)	67.1±4.5	54.8±5.2
Observation group (n=60)	65.9±4.8	50.3±4.1
*T*	-0.736	14.300
*P*	0.459	<0.05

While the control group had significant fluctuations in heart rate and blood pressure before anesthesia induction, revealing statistically significant differences between groups (P<0.05)(Table-III).

**Table-III:** Comparison of physiological indicators between groups.

Groups	Heart rate (times/min)			Diastolic pressure (mmHg)			Systolic pressure (mmHg)		
	Before health education	After health education	Before anesthesia induction	Before health education	After health education	Before anesthesia induction	Before health education	After health education	Before anesthesia induction
Control group (n=60)	81.6±10.1	80.7±10.4	86.6±10.9^a^	80.4±11.7	79.5±12.4	85.1±13.3^a^	121.6±14.5	120.3±13.4	129.7±14.9^a^
Observation group (n=60)	81.3±10.5	79.6±9.7	79.3±10.2^b^	80.9±12.9	79.1±13.2	78.9±12.9^b^	121.1±14.2	120.5±14.2	119.9±13.5^b^

***Note:*** Intra-group comparison before health education,

a P<0. 05; Inter-group comparison with the control group,

b P<0. 05.

Patients in the observation group experienced better perioperative psychological care effects and higher satisfaction degrees than the control group, with a statistically significant difference (P<0.05)(Table-IV).

**Table-IV:** Comparison of perioperative psychological care effects between groups.

Groups	Perioperative psychological care effects		
	Satisfied (12-18 points)	Relatively satisfied (7-11 points)	Dissatisfied (0-6 points)
Control group (n=60)	28	21	11
Observation group (n=60)	47	13	0

***Note:*** Comparison of perioperative psychological care effects between groups, *χ*^2^=7.956, P<0.05.

There was a statistically significant difference that the overall postoperative satisfaction degree of the observation group was higher than that of the control group (P<0.05)(Table-V).

**Table-V:** Comparison of postoperative satisfaction between groups.

Groups	Very satisfied (cases)	Satisfied (cases)	Dissatisfied (cases)	Overall degree of satisfaction [cases (%)]
Control group (n=60)	32	19	9	51
Observation group (n=60)	47	12	1	59

## DISCUSSION

Through comparative analysis of the data of 120 patients undergoing anterior cruciate ligament reconstruction, the study confirmed that the “Internet plus perioperative health education” model has significant advantages in improving patients’ psychological status, stabilizing physiological indicators and improving satisfaction. In the present study, after health education, both groups showed decreased SAS scores than those before, with statistically significant differences (p<0.05); and inter-group comparison revealed a lower degree of anxiety and more stable preoperative emotions in the observation group than those in the control group, with statistically significant difference (p<0.05). The observation group had a more stable heart rate and blood pressure, and the differences were statistically significant (p<0.05). In addition, according to the comparative analysis of perioperative psychological care effect scores and postoperative satisfaction between groups, there were statistically significant differences that the observation group showed better outcomes than the control group (p<0.05).

Compared with previous studies,^12^,^13^ the anxiety improvement effect of this study is more significant, which may be due to the continuous Internet interaction model (7-day intervention cycle) we adopted compared with its 3-day short-term intervention. Some studies show that the postoperative satisfaction of the national research report is 92%, slightly lower than 98.3% of this study.^14^ This difference may reflect the different acceptance and use habits of patients in different regions of mobile Internet health services. At the international comparison level,^15^ American scholars have achieved better physiological index stability by using virtual reality (VR) technology, but its per capita cost is as high as $230, far exceeding the WeChat platform solution.^16^ This cost-benefit difference is particularly critical in areas with limited medical resources.

This study is the first to systematically verify the effectiveness of a perioperative education model based on the WeChat platform in southwestern China, filling a regional research gap;^17^ Secondly, quantitative analysis reveals the stabilizing effect of Internet intervention on physiological parameters such as heart rate variability, which provides objective physiological evidence for digital health intervention;^18^ Most importantly, a low-cost and highly accessible digital education program has been developed that is suitable for the medical environment characteristics of second tier cities in China.^19^ From a clinical practice perspective, the findings of this study have multiple important implications:

- Providing an economically feasible solution to the common preoperative anxiety problem among surgical patients;
- Stabilizing vital signs may reduce the risk of intraoperative complications, which has potential value in improving surgical safety;^20^
- The high patient satisfaction rate of 98.3% not only reflects the advantages of this model in improving doctor-patient relationships, but also provides new ideas for improving the quality of hospital services.

The advantages of this study are mainly reflected in three aspects: strict surgical standardization process (all surgeries are completed by the same senior team), blind evaluation of results to avoid measurement bias, and a comprehensive evaluation system that simultaneously monitors psychological and physiological indicators. The rigorous design of these methodologies ensures the credibility of the research results.

This study is the first to validate WeChat based ACL patient education in China and quantify HRV stability. However, this study is a single center retrospective analysis, and a 6-month follow-up is not sufficient for long-term compliance assessment. A multicenter randomized controlled trial with 12-month follow-up is required in the future to validate the results of this study.

### Limitations

The single center design may affect the generalizability of the results; A 6-month follow-up period is insufficient for evaluating long-term functional recovery; No analysis was conducted on the differences in effectiveness among patients of different age groups. These limitations point the way for future research: conducting multicenter randomized controlled trials to validate the generalizability of the results; Extend the follow-up time to more than one year to evaluate long-term effects; Conduct cost-benefit analysis to provide a basis for health decision-making; Explore the applicability of this model in other orthopedic surgeries.

## CONCLUSIONS

The application of “Internet+health education” by nursing staff to perioperative patients with anterior cruciate ligament injury can not only reduce their preoperative anxiety, but also stabilize their physiological parameters and improve their satisfaction. It can provide practical guidance for exploring a new perioperative Internet+health education model in our hospital, and is worthy of clinical promotion and application.

### Authors’ Contributions:

**AX:** Conception and design, acquisition of data, analysis and interpretation of data.

**TL:** Drafting the article, revising it critically for important intellectual content and responsible for the accuracy and integrity of all data analyses.

**ZB and XC:** Accountable for data acquisition, methodological rigor, verified statistical validity and final approval of the version to be published.

All authors approved the final manuscript and assume public accountability

## References

[1] Song D, Ma Y, Zhang L, Ma Q (2022). Intermediate frequency electrotherapy stimulation to the medial femoris muscle for functional recovery of knee joint after anterior cruciate ligament reconstruction. Pak J Med Sci.

[2] Suh DK, Cho IY, Noh S, Yoon DJ, Jang KM (2023). Anatomical and Biomechanical Characteristics of the Anterolateral Ligament:A Descriptive Korean Cadaveric Study Using a Triaxial Accelerometer. Medicina (Kaunas).

[3] Cerciello S, Ollivier M, Kocaoglu B, Khakha RS, Seil R, ESSKA U45 Committee (2023). ACL surgical trends evolve in the last five years for young European surgeons:results of the survey among the U45 ESSKA members. Knee Surg Sports Traumatol Arthrosc.

[4] Musahl V, Engler ID, Nazzal EM, Dalton JF, Lucidi GA, Hughes JD (2022). Current trends in the anterior cruciate ligament part II:evaluation, surgical technique, prevention, and rehabilitation. Knee Surg Sports Traumatol Arthrosc.

[5] MARS Group (2019). Rehabilitation Predictors of Clinical Outcome Following Revision ACL Reconstruction in the MARS Cohort. J Bone Joint Surg Am.

[6] Wainwright TW, Gill M, McDonald DA, Middleton RG, Reed M, Sahota O (2020). Consensus statement for perioperative care in total hip replacement and total knee replacement surgery:Enhanced Recovery After Surgery (ERAS®) Society recommendations. Acta Orthop.

[7] Liu Q, Wang J, Han J, Zhang D (2022). Effect of Combining Operating Room Nursing Based on Clinical Quantitative Assessment with WeChat Health Education on Postoperative Complications and Quality of Life of Femoral Fracture Patients Undergoing Internal Fixation. J Healthc Eng.

[8] Collatuzzo G, Santucci C, Malvezzi M, La Vecchia C, Boffetta P, Negri E (2023). Trends in gastric cancer mortality 1990-2019 in 36 countries worldwide, with predictions to 2025, and incidence, overall and by subtype. Cancer Med.

[9] Wu Y, Wang X, Gao F, Liao J, Zeng J, Fan L (2023). Mobile nutrition and health management platform for perioperative recovery:an interdisciplinary research achievement using WeChat Applet. Front Med (Lausanne).

[10] Dunstan DA, Scott N (2020). Norms for Zung's Self-rating Anxiety Scale. BMC Psychiatry.

[11] Jiao Y (2000). Introduction of a scoring table for psychological care effect of perioperative patients. Chin J Nurs.

[12] Sun X, Li D, Guo Y, Chu HL, Sun XY, Yang YP (2020). Effects of mobile health intervention on early knee function after anterior cruciate ligament reconstruction:a prospective randomized controlled study. Zhonghua Wai Ke Za Zhi.

[13] Wang S, Shi W, Yang S, Cui J, Guo Q (2025). Marker-Less Navigation System for Anterior Cruciate Ligament Reconstruction with 3D Femoral Analysis and Arthroscopic Guidance. Bioengineering (Basel).

[14] Kietaibl S, Ahmed A, Afshari A, Albaladejo P, Aldecoa C, Barauskas G (2023). Management of severe peri-operative bleeding:Guidelines from the European Society of Anaesthesiology and Intensive Care:Second update 2022. Eur J Anaesthesiol.

[15] Levine Obe R, Stillman-Lowe C (2024). Health education. Br Dent J.

[16] Nukuto K, Hoshino Y, Kataoka K, Kuroda R (2023). Current development in surgical techniques, graft selection and additional procedures for anterior cruciate ligament injury:a path towards anatomic restoration and improved clinical outcomes-a narrative review. Ann Jt.

[17] Mhaskar VA, Jain Y, Soni P, Fiske R, Maheshwari J (2021). How Important is the Tunnel Position in Outcomes Post-ACL Reconstruction:A 3D CT-Based Study. Indian J Orthop.

[18] Wen Y, Guo X, Sun G, Liu Q, Qin D (2023). Application effect of orthopedic specialist nurse?led “Internet+medical health”intervention in patients with anterior cruciate ligament reconstruction. Chin Nurs Res.

[19] Tang H, Xiao YF, Liu WJ, Meng JH, Wu YM, Xiong YL (2024). Preferences in anterior cruciate ligament reconstruction:A survey among orthopedic surgeons in China. Medicine (Baltimore).

[20] Springer B, Bechler U, Koller U, Windhager R, Waldstein W (2020). Online Videos Provide Poor Information Quality, Reliability, and Accuracy Regarding Rehabilitation and Return to Sport After Anterior Cruciate Ligament Reconstruction. Arthroscopy.

